# Isolation and Characterization of Thermophilic Bacteria from a Hot Spring in the State of Hidalgo, Mexico, and Geochemical Analysis of the Thermal Water

**DOI:** 10.3390/microorganisms12061066

**Published:** 2024-05-24

**Authors:** Rosangel Ortega-Villar, Adelfo Escalante, Fernando Astudillo-Melgar, Liliana Lizárraga-Mendiola, Gabriela A. Vázquez-Rodríguez, María Eugenia Hidalgo-Lara, Claudia Coronel-Olivares

**Affiliations:** 1 Área Académica de Química, Universidad Autónoma del Estado de Hidalgo, Carretera Pachuca-Tulancingo Km. 4.5, Mineral de la Reforma 42184, Hidalgo, Mexico; or214835@uaeh.edu.mx (R.O.-V.);; 2 Departamento de Ingeniería Celular y Biocatálisis, Instituto de Biotecnología, Universidad Nacional Autónoma de México, Cuernavaca 62210, Morelos, Mexico; 3 Área Académica de Ingeniería y Arquitectura, Universidad Autónoma del Estado de Hidalgo, Carretera Pachuca-Tulancingo Km. 4.5, Mineral de la Reforma 42184, Hidalgo, Mexico; 4 Departamento de Biotecnología y Bioingeniería, CINVESTAV, San Pedro Zacatenco, Mexico City 2508, Mexico

**Keywords:** thermal water, hydro-geochemistry, enzymes, thermophilic bacteria, firmicutes, deinococcus-thermus

## Abstract

Hot springs worldwide can be a source of extremophilic microorganisms of biotechnological interest. In this study, samplings of a hot spring in Hidalgo, Mexico, were conducted to isolate, identify, and characterize morphologically, biochemically, and molecularly those bacterial strains with potential industrial applications. In addition, a physicochemical and geochemical examination of the hot spring was conducted to fully understand the study region and its potential connection to the strains discovered. The hot spring was classified as sulfate-calcic according to the Piper Diagram; the hydrogeochemical analysis showed the possible interactions between minerals and water. Eighteen bacterial strains were isolated with optimal growth temperatures from 50 to 55 °C. All strains are Gram-positive, the majority having a rod shape, and one a round shape, and 17 produce endospores. Hydrolysis tests on cellulose, pectin, and xylan agar plates demonstrated enzymatic activity in some of the strains. Molecular identification through the 16S rDNA gene allowed classification of 17 strains within the Phylum Firmicutes and one within Deinococcus-Thermus. The bacterial strains were associated with the genera *Anoxybacillus*, *Bacillus*, *Anerunibacillus*, *Paenibacillus*, and *Deinococcus*, indicating a diversity of bacterial strains with potential industrial applications.

## 1. Introduction

Extremophilic microorganisms thrive in extreme environments, due to their adaptive capacity and stress tolerance in operating metabolically and biochemically under harsh conditions [1,2,3]. Most extremophilic organisms are unicellular, spanning all three life domains: Archaea, Eucarya, and Bacteria [4,5]. These organisms are resilient in extreme conditions, such as acidic or alkaline pH, high pressures, high salt concentrations, ionizing radiation, as well as low or high temperatures [6].

Microorganisms thriving at elevated temperatures are called thermophiles [7], which can inhabit such environments due to their molecular properties, metabolic versatility, size, and inherent physical and chemical characteristics [7,8]. Based on their optimal growth temperature, thermophiles can be classified as follows: thermophiles (50–60 °C), extreme thermophiles (60–80 °C), and hyperthermophiles (80–110 °C) [9]. The microbiological biodiversity of thermophiles can offer insights into community organization and their potential applications in industrial processes [10], as thermophiles are considered a source of thermostable enzymes or thermo-enzymes [9]. These enzymes are of interest in industries operating under extreme environmental conditions, as they exhibit higher tolerance and resistance than enzymes of mesophilic organisms [11,12]; moreover, some thermo-enzymes remain active in detergents and solvents [13]. Examples of thermostable enzymes include xylanases, proteases, amylases, peroxidases, glucose isomerases, lipases, cellulases, pectinases, and DNA restriction enzymes [4,8,9,14].

The diversity of thermophiles has been studied in various locations worldwide [10], primarily in areas heated solely by solar radiation or in geothermal zones [15]. Hot springs, for instance, represent one of Earth’s extreme environments [16], associated with regions of senile or inactive volcanic activity [17], where temperature can be attributed to geothermal energy, exothermic chemical reactions, or radioactive decay [18]. Thus, geothermal systems provide habitats for thermophiles, and their physiological and morphological diversity may be crucial to evolution in geothermal reservoirs, while water-rock interactions may be an important factor [19]. Physicochemical parameters, such as pH, temperature, electrical conductivity, total dissolved solids, ions, and heavy metals, can influence the quality of thermal water. For instance, elevated temperatures lead to an increased interaction with surrounding rocks and minerals [18]; thus, the chemistry of spring water is linked to its geological background [20]. In turn, in thermal environments, microbial diversity can be influenced by temperature, pH, and the chemical composition of water [16,19,21].

Studies have been conducted in various hot springs worldwide, where thermophilic bacteria with potential industrial applications have been isolated. For instance, the genera *Bacillus* and *Geobacillus* have been reported in hot springs with temperatures ranging from 42 to 82 °C in the Czech Republic [22]. In Algeria, the genera *Bacillus*, *Anoxybacillus*, *Aeribacillus*, *Aneurinibacillus*, *Brevibacillus*, *Geobacillus*, and *Thermus* were reported in hot springs with temperatures ranging from 40 to 73 °C [10]. In India, the genus *Bacillus* was reported in hot springs with temperatures of 50, 65, and 95 °C [23].

In this study, bacterial strains were isolated from the hot spring of Santa María Amajac, located in the Neovolcanic Range or Transversal Volcanic Axis, which extends across twelve states of Mexico, including Hidalgo State [24]. Firstly, the hydrogeochemical characteristics of the study area were identified. Secondly, microbiological diversity was assessed through the colonial, morphological, and biochemical characterization of the isolated strains, including their potential as sources of thermostable enzymes, like cellulase, pectinase, and xylanase, for industrial processes. Finally, molecular identification of the strains was conducted to reveal their place in the phylogenetic tree.

## 2. Materials and Methods

### 2.1. Sampling and Physicochemical Characterization of Water

Water samples were collected from two different areas of the hot spring spa in Santa María Amajac, Hidalgo, Mexico, with the following coordinates: longitude −98.742222 and latitude 20.323889. Samples identified as Z1 were taken from an open water well subject to contamination by fauna, flora, and anthropogenic factors (well 1). In contrast, samples identified as Z2 were extracted from a well, covered and sealed with a concrete slab, without contact with the public or spa staff (well 2). In situ measurements of temperature, pH, electrical conductivity (EC), dissolved oxygen (DO), total dissolved solids (TDSs), oxidation-reduction potential (ORP), and salinity were performed using the HANNA HI 9828 portable calibrated multiparameter instrument (Woonsocket, RI, USA). Water samples for microbiological analyses were collected in sterile 100-mL Whirl-Pak^®^ bags (Nasco^TM^, Fort Atkinson, WI, USA), transported in an insulated container to maintain their temperature, and processed on the same day. Additionally, a 1-L water sample was taken exclusively from well 2 in a sterile container for physicochemical analysis, which included pH, Ca^2+^, Mg^2+^, Na^+^, K^+^, PO_4_^−^, NO_3_^−^, CO_3_^2−^, HCO_3_^−^, Cl^−^, SO_4_^2−^, hardness, electrical conductivity, and effective salinity. This sample was delivered to the laboratory Análisis Técnicos S. A. de C. V., endorsed by EMA (Mexican Testing Laboratory, A-0618-060/15). This analysis was conducted solely to characterize the hydro-geochemistry of the site.

### 2.2. Hydrogeochemical Analysis

The hydrogeochemical analysis of the thermal water was conducted using a Piper diagram generated with Easy-Quim.4 software [25]. This diagram illustrates the ratio between cations and anions represented by two triangles and a rhombus. The left triangle presents the cations (Ca^2+^, Mg^2+^, Na^+^, K^+^), and the right triangle the anions (Cl^−^, SO_4_^2−^, CO_3_^2−^, HCO_3_^-^), while the rhombus displays the proportion of both [26]. Cation readings increased from right to left, anions increased from left to right, and in both cases percentages increased from bottom to top, showing the predominant chemical groups in the thermal waters.

Geochemical modeling of the water was also performed using the free software PHREEQC Version 3 (USGS, USA) [27]. The values of pH, chemical conductivity, sample temperature at the analysis (26.1 °C), and ionic concentrations were utilized. Ion exchange and surface complexation reactions were modeled using “aqueous speciation” and “phase equilibrium” options. The wateq4f database was selected to analyze the correlation between ionic strength and water chemical composition, for determination of the mineral phases potentially involved in geochemical exchange.

### 2.3. Isolation and Characterization of the Isolated Strains

Samples of thermal water (Z1 and Z2) were cultured using the membrane filtration technique with nitrocellulose membranes of 0.45 µm diameter pore (Millipore Corporation, Billerica, MA, USA), employing serial decimal dilutions (10^−1^, 10^−2^, and 10^−3^) in sterile 0.85% NaCl solution. The membranes from the filtration of direct samples and dilutions were placed on nutritive agar (DIBICO^®^, Cuautitlán Izcalli, Mexico) supplemented with bacteriological agar (Becton Dickinson, Detroit, MI, USA) and prepared with thermal water previously sterilized by filtration. All media were sterilized at 121 °C for 15 min in an electric autoclave (All American 1941X, Manitowoc, WI, USA). Plates were incubated for 24 h at 50 °C in a Lab-Line Imperial III incubator (Melrose Park, IL, USA). Subsequently, various colonies showing growth separately from each other were selected. To obtain axenic cultures, these colonies were recultured on the same medium under identical incubation conditions. A code was assigned to each isolated strain.

#### 2.3.1. Microscopic Observation

Gram staining was performed on each isolated strain following the Smith and Hussey technique [28]. Additionally, spore staining was conducted using the Schaeffer–Fulton method [29]. Samples were observed at 100× magnification using a binocular microscope B3-220 PL (MOTIC^®^, Barcelona, Spain).

#### 2.3.2. Morphological Characterization

The morphological characterization of strains on both solid and liquid media was carried out, according to Smith [30]. For tests on solid media, strains were streaked on agar plates by square streak, inoculated by deep stabbing into vertical agar tubes, and straight streaked on slant tubes filled with nutrient agar (DIBICO^®^, Cuautitlán Izcalli, Mexico), supplemented with bacteriological agar (Becton Dickinson, Detroit, MI, USA). For characterization in liquid media, strains were inoculated into nutrient broth (Becton Dickinson, Cuautitlán Izcalli, Mexico). Both plates and tubes were incubated at 50 °C for 24 h. Colony counting was performed using a Quebec-type colony counter (Felisa, Zapopan, Mexico) to visualize growth and colony characterization.

#### 2.3.3. Physiological Characterization

To determine the temperature and pH growth ranges of strains, they were incubated at different temperatures (25, 37, 50, and 70 °C) and pH values (5, 6, 7, 8, and 9) adjusted with 1 M solutions of NaOH or HCl. Salt tolerance tests were also conducted at 2%, 5%, and 7% (*w*/*v*) NaCl concentrations. All tests were performed in nutrient broth (Bioxon^®^, Atizapán de Zaragoza, Mexico) and incubated for 24 h at 50 °C, except for temperature tests [31].

#### 2.3.4. Biochemical Characterization

For biochemical tests, up to 24-h cultures were used. Unless otherwise specified, both plates and tubes were incubated at 50 °C for 24 h. Two certified control strains, *Escherichia coli* CDBB-B-1010 and *Bacillus subtilis* CDBB-1349, were used to validate all tests.

#### Sugar Fermentation, Hydrogen Sulfide, and Gas Production

Strains were inoculated into triple sugar iron agar (Becton Dickinson, Cuautitlán Izcalli, Mexico). Glucose, lactose, and sucrose fermentation was observed by color change in the medium along the tube (acidification or alkalinization of the medium), H_2_S production by black coloration and gas accumulation, both at the tube’s base [32].

#### Indole Test

Strains were inoculated into SIM medium (Becton Dickinson, Mexico). After incubation, a few drops of Kovac’s indole reagent (Sigma-Aldrich, Saint Louis, MO, USA) were added. The presence of red color indicates tryptophan hydrolysis [33].

#### Catalase Activity

Nutrient agar plates supplemented with bacteriological agar (DIBICO^®^, Cuautitlán Izcalli, Mexico; Becton Dickinson, Detroit, MI, USA, respectively) were inoculated with 30% hydrogen peroxide (H_2_O_2_). The test is considered positive if effervescence occurs, which results from the decomposition of hydrogen peroxide into water and oxygen [34].

#### Casein Hydrolysis

Strains were inoculated into skim milk agar (Fluka, Buchs, Switzerland). The presence of proteases is observed due to the formation of clumps in the medium [35].

#### Starch Hydrolysis

Strains were inoculated into a medium containing beef extract (Becton Dickinson, Cuautitlán Izcalli, Mexico) 3 g/L, soluble starch 10 g/L (MEYER, Tláhuac, Mexico), and agar (Hycel, Zapopan, Mexico) 12 g/L, with pH adjusted to 7.5 using 1 M NaOH or HCl. Amylolytic activity was detected by the formation of a blue/black color around bacterial growth upon addition of Gram’s iodine (Merck KGaA, Darmstadt, Germany) [36].

#### Gelatin Hydrolysis

A medium containing peptone (Becton Dickinson, Sparks, MD, USA) 5 g/L, beef extract (Becton Dickinson, Cuautitlán Izcalli, Mexico) 3 g/L, and gelatin (Fermont, Monterrey, Mexico) 120 g/L, at pH 6.8, was used. Tubes containing the medium were placed on ice for 30 min. Strains were inoculated by deep stabbing and incubated at 25 °C for 7 days. Gelatinase activity is observed by liquefaction of the medium. Before confirming the result, tubes were kept at 2–8 °C for 30 min to ensure that liquefaction was due to gelatinase activity [37].

#### Cellulose, Pectin, and Xylan Hydrolysis

For hydrolysis tests, strains were incubated for 24 h at 50 °C in a medium modified from Santiago-Hernández et al. [38]. The base medium was formulated as follows: phosphate buffer (100 mM, pH 7), prepared with NaH_2_PO_4_ and Na_2_HPO_4_·7H_2_O, with pH adjusted using a mineral medium containing NaCl 5.5 g/L; granular (NH_4_)_2_SO_4_ 2.5 g/L; CaCl_2_ 0.1 g/L; MgSO_4_ 0.1 g/L; 0.2% yeast extract (Sigma-Aldrich, Bengalore, India), and 2% agar (Sigma-Aldrich, Madrid, Spain).

Additionally, for cellulose hydrolysis, carboxymethyl cellulose (Sigma-Aldrich, St. Louis, MO, USA) was added to the base medium described above. Twenty-four hours later, the modified method by Kasana et al. [39] was used. The plates were flooded with 1% Congo red (Sigma-Aldrich, St. Louis, MO, USA), and then washed with 1 M NaCl. The formation of a halo around the culture indicates cellulose hydrolysis. 

The same base medium for pectin hydrolysis was used but supplemented with 1% esterified citrus pectin potassium salt (Sigma-Aldrich, St. Louis, MO, USA) according to a method modified from Villalba et al. [40]. Cultures were washed with Tris–HCl buffer, prepared with Tris (BIO-RAD, Hercules, CA, USA) and hydrochloric acid, followed by washing with acetate buffer, prepared with anhydrous sodium acetate, and adjusting the pH with glacial acetic acid, and a final wash with ruthenium red (Sigma-Aldrich, St. Louis, MO, USA). The formation of a halo around the colony was interpreted as positive for pectin hydrolysis.

Lastly, for xylan hydrolysis, 1% 4-O-methyl-D-glucurono-D-xylan with remazol brilliant blue (Sigma-Aldrich, St. Louis, MO, USA) and 1% xylan (BIOpHORETICS, Sparks, NV, USA) was added to the base medium described above. According to a method modified from Cayetano-Cruz et al. [41], the formation of a white halo around the colony was considered positive for xylan hydrolysis.

### 2.4. Molecular Identification of the Isolated Strains

The isolated strains were characterized molecularly by sequencing the 16S rDNA gene. Bacterial DNA extraction was conducted using the Quick-g DNA™ Miniprep Kit from Zymo Research (Irvine, CA, USA), following the manufacturer’s instructions. The quality of extracted DNA was analyzed using standard procedures using 1% agarose gel electrophoresis. DNA quantification was performed using a NanoDrop 2000c instrument from Thermo Scientific (Wilmington, DE, USA).

The 16S rDNA gene was amplified by PCR using a GeneAmp PCR System 9600 thermocycler (Perkin Elmer, Singapore) with the primers fd1 (forward) (5′ AGAGTTTGATCCTGGCTCAG3′) and rd1 (reverse) (5′AAGGAGGTGATCCAGCC3′) [42]. The amplification conditions were as follows: 95 °C for 5 min (1 cycle), 95 °C for 30 s, 55 °C for 30 s, and 72 °C for 1.5 min, with a final extension at 72 °C for 5 min. Using both primers’ set pairs allowed the amplification of a 1.5 Kb product corresponding to the size of the 16S rDNA gene.

As mentioned above, the PCR amplification product size was confirmed on an aga-rose gel and purified using the GeneJET PCR Purification Kit from ThermoFisher Scientific (Vilnius, Lithuania) following the manufacturer specifications and DNA concentration quantified as above. 

Samples were prepared according to the conditions requested by the DNA Synthesis and Sequencing Unit of the Institute of Biotechnology (UNAM, Mexico). Resulting sequences were analyzed using SnapGene 7.0.2 software to assemble the 16S rDNA gene from the obtained sequences. Each assembled sequence was compared with the GenBank database using the NCBI Nucleotide BLAST^®^ 2.13.0 application [43], which provides each sequence’s taxonomical identity or the closest taxon. A phylogenetic analysis of the 16S rDNA sequences obtained for isolated bacteria and reference sequences retrieved from the GenBank database was conducted using MEGA 11 software [44]. An initial sequence alignment was performed using Muscle program with the predefined parameters. The resulting alignment was used to build a distance matrix with the Jukes–Cantor model and a boot-strapping of *n* = 1000 replicates. Finally, the resulting distance matrix was used to construct a phylogenetic (phenetic) tree using the maximum likelihood method with the Jukes–Cantor model and a bootstrapping of *n* = 1000 replicates.

## 3. Results

### 3.1. Physicochemical Characterization of Hot Spring Water

Table 1 exhibits the results of the in situ physicochemical characterization of the Santa María Amajac hot spring, for both sampling sites (Z1 and Z2). The linear distance between the two sampling sites is approximately 168 m, which might be considered as close enough to produce similar physicochemical characteristics in the two samples. However, the values of DO, pH, EC, TDS, and salinity were higher in Z1 than in Z2, while temperature and ORP values were higher in Z2 than in Z1.

The physicochemical characterization of thermal water (Z2) is shown in Table 2. It was detected that the major cation was Ca^2+^; however, by far, the most abundant component was SO_4_^2−^, which was found in concentrations approaching the highest values found in the consulted reports [17,18,19]. The EC values reported by the laboratory were slightly different (by 127 µS/cm) from those measured in situ by the field multiparameter sensor.

### 3.2. Hydrogeochemical Analysis

**Figure 1:**
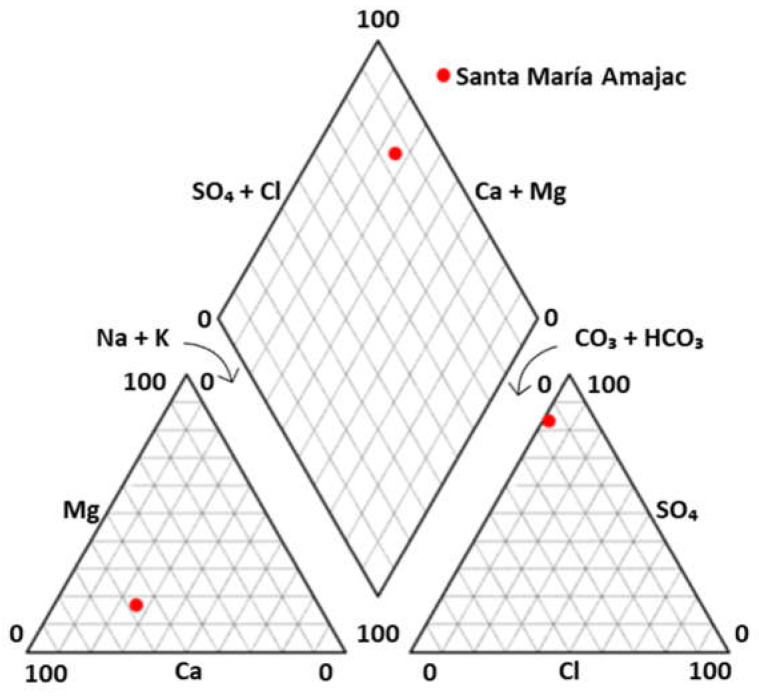
Piper diagram of the thermal water from site Z2 in Santa María Amajac.

The hydrogeochemical analysis with the existing proportion between major cations (Na^+^ + K^+^; Na^+^; K^+^; Ca^2+^; Mg^2+^) and anions (HCO_3_^−^; SO_4_^2−^; Cl^−^; NO_3_^−^; Cl^−^) in Z2 sample can be visualized in the resulting Piper diagram (Figure 1). It is worth noting that the value of the total dissolved solids measured in situ at site Z2 was also taken for the analysis. As presented before, the major cation was Ca^2+^ (57.33%), followed by Na^+^ + K^+^ (25.80%), and Mg^2+^ (16.87%), while the major anions were SO_4_^2−^ (83.39%), and HCO_3_^−^ (14.93%). Consequently, Z2 thermal waters can be classified as sulfate-calcic waters (83.39% SO_4_^2−^ + 57.33% Ca^2+^) (Figure 1). Although this work did not analyze the mineralogy of the study area, rock groups such as calcareous and clastic rocks (with a predominant presence of carbonates, sulfates, and clay minerals), as well as volcanic rocks (rich in quartz, feldspars, and calcium-rich plagioclases) could lead to the hydrogeochemical characteristics signaled [45].

**Figure 2:**
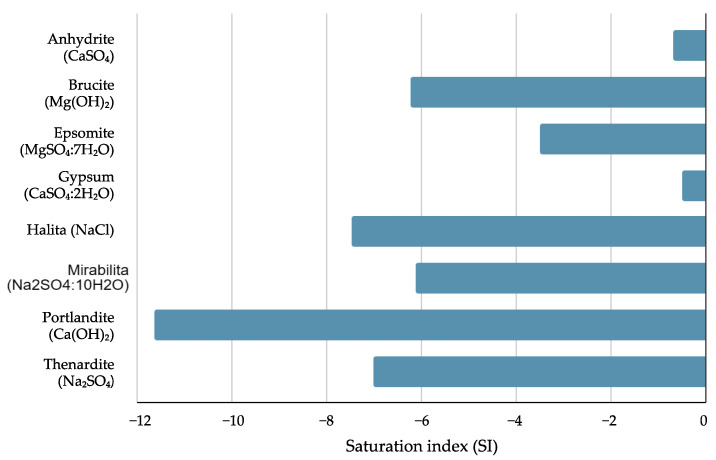
Geochemistry of the thermal water from site Z2 in Santa María Amajac modeled by PHREEQC.

The results of the geochemical analysis show the mineral composition as compared to the wateq4f database of PHREEQC software (Figure 2).

Figure 2 highlights that all minerals associated with the ions present in the analyzed water sample are sub-saturated (saturation index, SI < 0), indicating the possibility of geochemical interactions of the thermal water with the local geology. This geochemical behavior could favor mineral dissolution of volcanic rocks (predominantly) and carbonate and clastic rocks (secondarily), hence the presence of these ions in solution.

### 3.3. Characterization of the Isolated Strains

The membrane filtration technique allowed for the initial observation of a diversity of colony-forming units (CFUs), which were randomly selected. From Z1, seven CFUs were isolated, exhibiting cultural differences on agar plates. From Z2, eleven bacterial strains were isolated, showing even greater cultural diversity.

#### 3.3.1. Microscopic Observation

**Figure A1:**
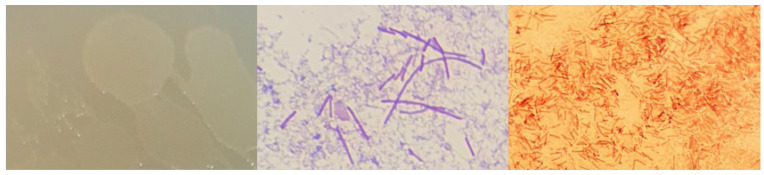
Z1-1 strain. Agar plate morphology (**left**), cell morphology and Gram staining (**center**), and endospores (**right**).

**Figure A2:**
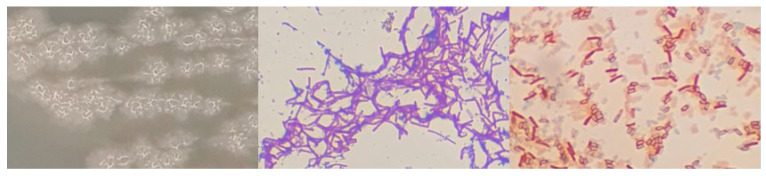
Z1-2 strain. Agar plate morphology (**left**), cell morphology and Gram staining (**center**), and endospores (**right**).

**Figure A3:**
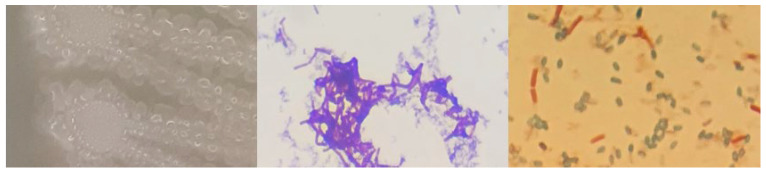
Z1-3 strain. Agar plate morphology (**left**), cell morphology and Gram staining (**center**), and endospores (**right**).

**Figure A4:**
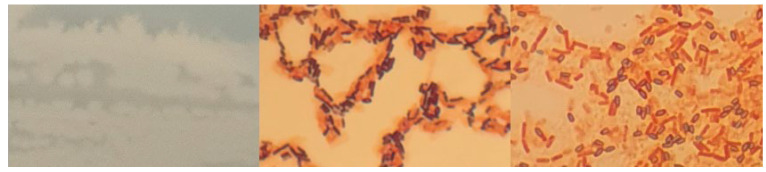
Z1-4 strain. Agar plate morphology (**left**), cell morphology and Gram staining (**center**), and endospores (**right**).

**Figure A5:**
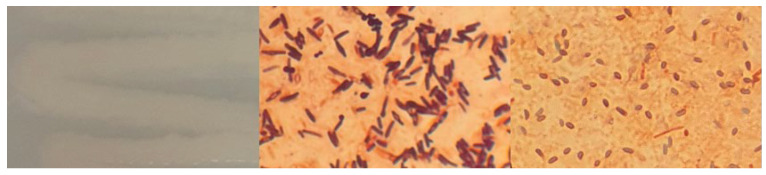
Z1-5 strain. Agar plate morphology (**left**), cell morphology and Gram staining (**center**), and endospores (**right**).

**Figure A6:**
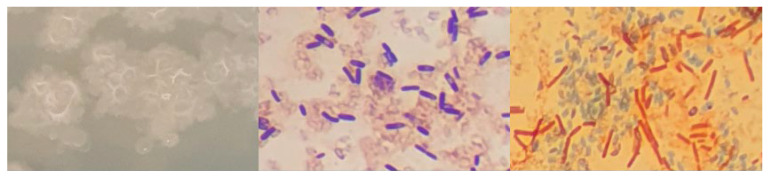
Z1-6 strain. Agar plate morphology (**left**), cell morphology and Gram staining (**center**), and endospores (**right**).

**Figure A7:**
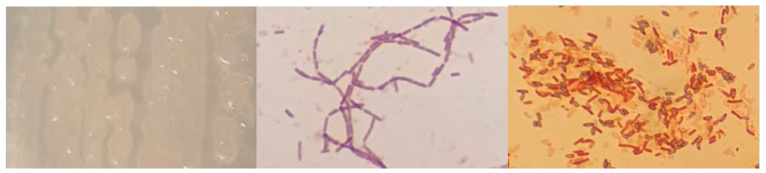
Z1-7 strain. Agar plate morphology (**left**), cell morphology and Gram staining (**center**), and endospores (**right**).

**Figure A8:**
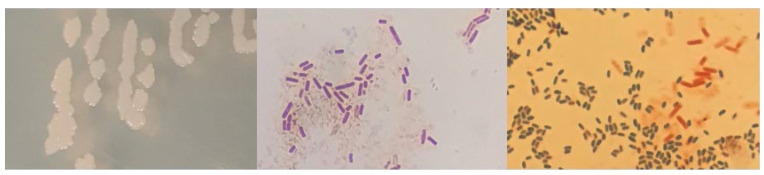
Z2-1 strain. Agar plate morphology (**left**), cell morphology and Gram staining (**center**), and endospores (**right**).

**Figure A9:**
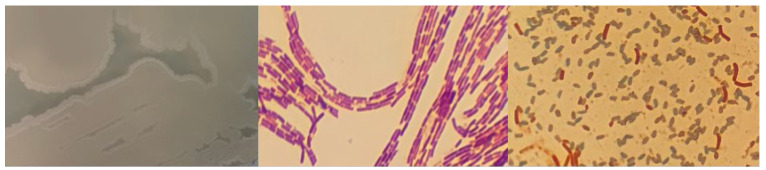
Z2-2 strain. Agar plate morphology (**left**), cell morphology and Gram staining (**center**), and endospores (**right**).

**Figure A10:**
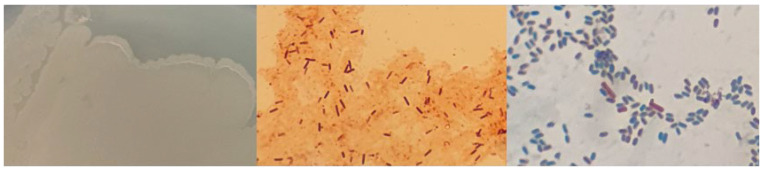
Z2-3 strain. Agar plate morphology (**left**), cell morphology and Gram staining (**center**), and endospores (**right**).

**Figure A11:**
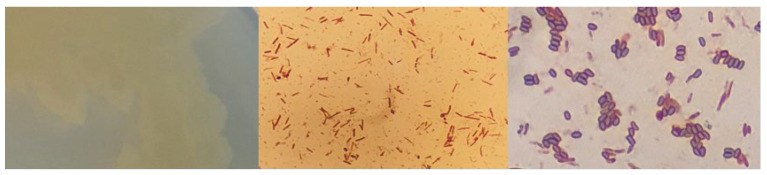
Z2-4 strain. Agar plate morphology (**left**), cell morphology and Gram staining (**center**), and endospores (**right**).

**Figure A12:**
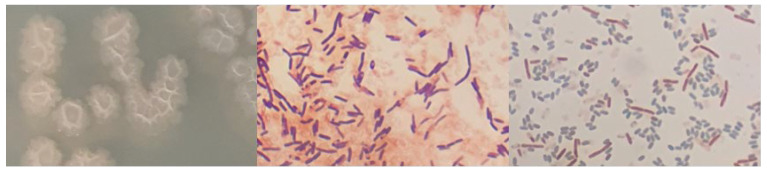
Z2-5 strain. Agar plate morphology (**left**), cell morphology and Gram staining (**center**), and endospores (**right**).

**Figure A13:**
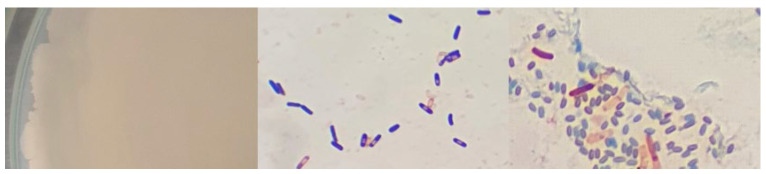
Z2-6 strain. Agar plate morphology (**left**), cell morphology and Gram staining (**center**), and endospores (**right**).

**Figure A14:**
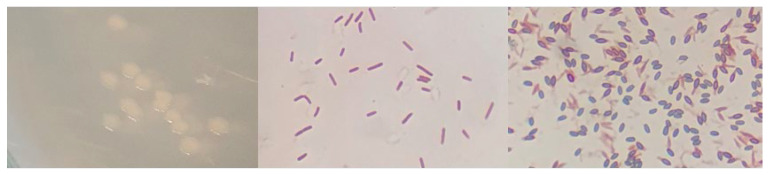
Z2-7 strain. Agar plate morphology (**left**), cell morphology and Gram staining (**center**), and endospores (**right**).

**Figure A15:**
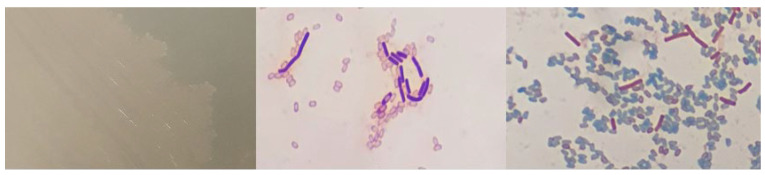
Z2-8 strain. Agar plate morphology (**left**), cell morphology and Gram staining (**center**), and endospores (**right**).

**Figure A16:**
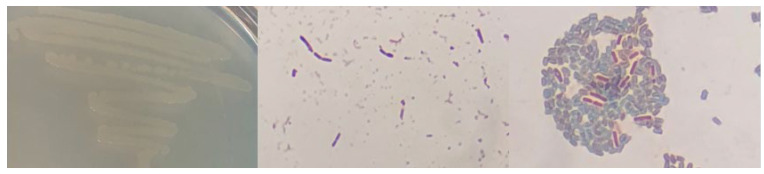
Z2-9 strain. Agar plate morphology (**left**), cell morphology and Gram staining (**center**), and endospores (**right**).

**Figure A17:**
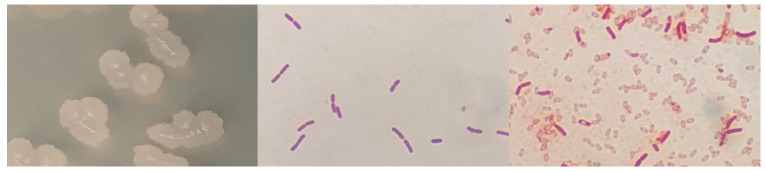
Z2-10 strain. Agar plate morphology (**left**), cell morphology and Gram staining (**center**), and endospores (**right**).

**Figure A18:**
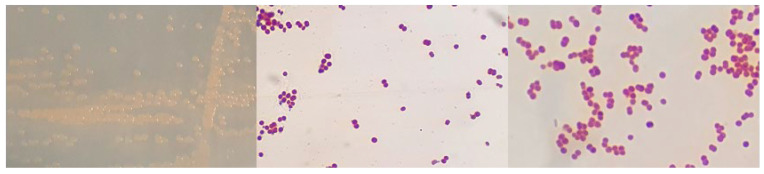
Z2-11 strain. Agar plate morphology (**left**), cell morphology and Gram staining (**center**), and negative to endospores strain (**right**).

The morphology of the seven strains isolated from site Z1 and the eleven strains from site Z2 is shown in the Appendix A section. Both Gram staining and endospore staining were conducted with these cultures. All strains extracted from Z1(Z1-1 to Z1-7) exhibited rod morphology and were Gram-positive (Figure A1, Figure A2, Figure A3, Figure A4, Figure A5, Figure A6 and Figure A7, center), with the ability to produce endospores (Figure A1, Figure A2, Figure A3, Figure A4, Figure A5, Figure A6 and Figure A7, right). Greater diversity was observed in Z2, as ten of the strains (Z2-1 to Z2-10) exhibited rod morphology (Figure A8, Figure A9, Figure A10, Figure A11, Figure A12, Figure A13, Figure A14, Figure A15, Figure A16 and Figure A17, center), while one (Z2-11) displayed coccus morphology (Figure A18, center). Additionally, all strains isolated from Z2 were Gram-positive (Figure A8, Figure A9, Figure A10, Figure A11, Figure A12, Figure A13, Figure A14, Figure A15, Figure A16, Figure A17 and Figure A18, center), and most produced endospores (Figure A8, Figure A9, Figure A10, Figure A11, Figure A12, Figure A13, Figure A14, Figure A15, Figure A16 and Figure A17, right), except for strain Z2-11 (Figure A18) (shown in the Appendix A Section). 

#### 3.3.2. Morphological Characterization of the Isolated Strains

Cultural characteristics of both solid (plates and tubes) and liquid media are presented in Table 3. On agar plates (Figure A1, Figure A2, Figure A3, Figure A4, Figure A5, Figure A6, Figure A7, Figure A8, Figure A9, Figure A10, Figure A11, Figure A12, Figure A13, Figure A14, Figure A15, Figure A16, Figure A17 and Figure A18, left), strains with amoeboid morphology predominated, featuring lobed edges, flat elevation, and a butyrous texture. Most isolated strains were white or beige, except for one yellow-colored strain (Z2-4) and one orange-colored strain (Z2-11). Papillate morphology was predominant in agar deep tubes, while echinulate morphology predominated in agar slant tubes. In liquid media, surface growth was predominantly membranous with slight or transient opacity, accompanied by viscid and scanty sediment.

#### 3.3.3. Physiological Characterization of the Isolated Strains

The growth under different temperatures and pH values, as well as the results of NaCl tolerance tests, are shown in Table 4. On the one hand, most strains exhibited growth at 25 °C, while all showed growth, with some producing a significant amount of biomass, at 37 °C. As the temperature increased, a higher biomass was observed for samples extracted from both sites, Z1 and Z2. Additionally, optimal growth for different strains was observed at 50 °C for some and at 60 °C for others. On the other hand, pH tolerance tests revealed that some strains were inhibited or showed scanty biomass at pH 6 and 5. For most strains, biomass increased starting from pH 7, with some increasing in biomass at pH 9. Finally, the strains showed tolerance to NaCl at concentrations of 2% and 5%, but when the concentration was increased to 7%, most strains exhibited scanty biomass.

#### 3.3.4. Biochemical Characterization of the Isolated Strains

The results of the biochemical tests conducted are presented in Table 5. Most strains fermented glucose, while strains Z1-3, Z2-5, and Z2-7 are the only strains that could also ferment lactose and sucrose. Five strains did not ferment any of the three carbohydrates. Only strain Z2-7 could produce hydrogen sulfide, and none produced gas attributed to carbohydrate fermentation. All strains were negative for the indole test. Regarding catalase activity, all strains tested positive except for strain Z1-1.

Most strains could hydrolyze casein, except for strain Z2-2. Strains Z1-2, Z1-6, Z1-7, Z2-1, Z2-2, Z2-5, Z2-6, and Z2-10 hydrolyzed starch. Most strains hydrolyzed gelatin, except for Z1-1, Z2-4, and Z2-11.

Most strains hydrolyzed cellulose, except for Z1-1, Z1-5, Z2-4, and Z2-1. Only strains Z1-3, Z1-4, Z1-6, Z1-7, and Z2-5 hydrolyzed pectin. Strains Z2-1, Z2-2, Z2-3, Z2-6, and Z2-10 were the only strains that could hydrolyzed xylan.

### 3.4. Molecular Identification of the Isolated Strains

The molecular identification results from database at GenBank are shown in Table 6. Overall, the sequence similarity of all strains to a known taxon is above 97%.

**Figure 3:**
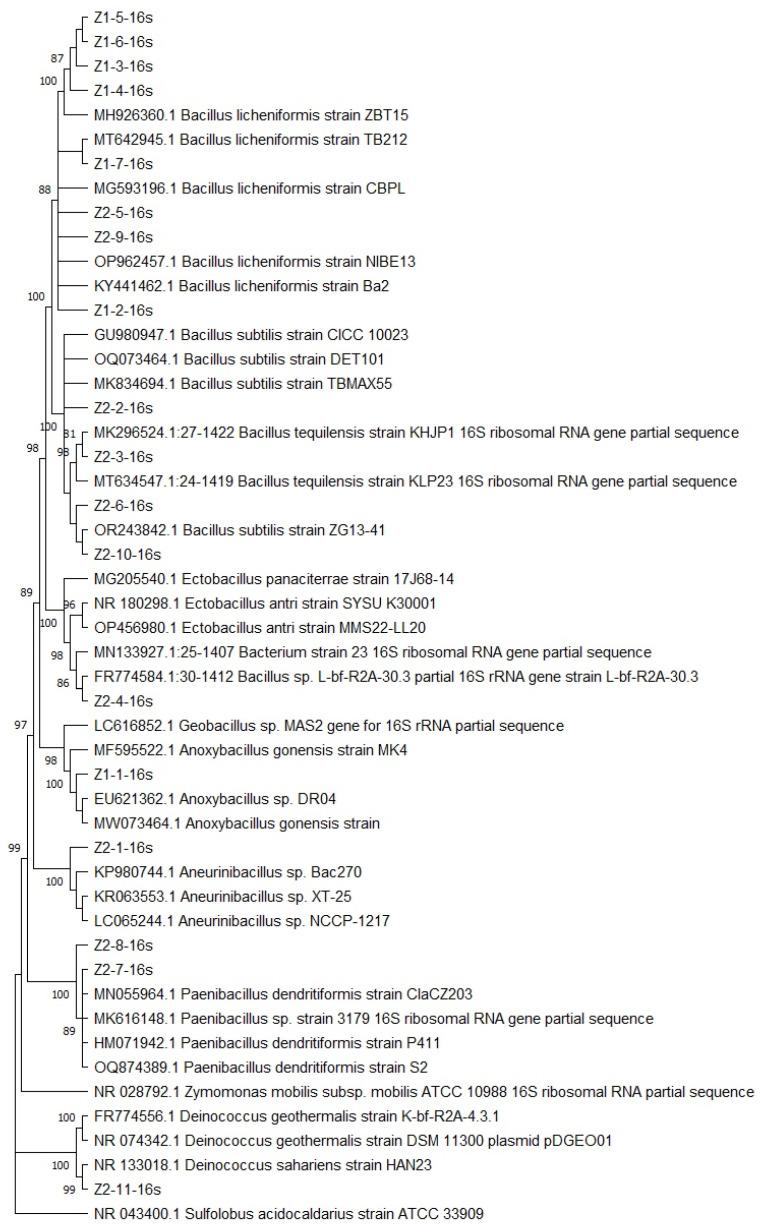
Phylogenetic tree of the isolated strains using the maximum likelihood method with the Jukes–Cantor model and Bootstrapping of *n* = 1000 replicates. The percentage of 1000 boot-strap samplings supporting each topological element in the maximum likelihood analysis is indicated. The 16S rDNA sequence of *Sulfolobus acidocaldarius* ATCC 33909 served as outgroup. The percentage of 1000 bootstrap samplings supporting each topological element in the neighbor-joining analysis is indicated. No values are given for groups with bootstrap values less than 80%.

The identity of the 18 analyzed strains indicated that 17 correspond to Gram-positive bacteria (Phylum Firmicutes), except for strain Z2-11, identified as closely related to *Deinococcus sahariens*, which belongs to the Phylum Deinococcus-Thermus. Among the isolates belonging to the Phylum Firmicutes, strain Z1-1 was identified as *Anoxybacillus gonensis* with an identity percentage of 99.86%; strains Z1-2, Z1-3, Z1-4, Z1-5, Z1-6, Z1-7, Z2-5, and Z2-9 were identified as *Bacillus licheniformis* with identity percentages ranging from 97% to 100%; strains Z2-6 and Z2-10 were related to *Bacillus subtilis* with identity percentages of 100% and 99.93%, respectively. Strain Z2-1 was associated with the taxon *Aneurinibacillus* sp. with an identity percentage of 99.78%. Strains Z2-2 and Z2-3 were related to *Bacillus tequilensis* with 100% and 99.78% identity percentages, respectively. Strains Z2-7 and Z2-8 were associated with the species *Paenibacillus dendritiformis*, with identity percentages of 100% and 99.44%, respectively. The Blast analysis of the 16S DNA sequence of the isolate Z2-4 resulted in an identity of 99.86% with a Bacterium isolate (GenBank MN133927.1), 99.86% of identity with the *Ectobacillus* sp. JY-23 chromosome complete sequence (GenBank CP095462.1), and 99.87% of identity with a *Bacillus* sp. (GenBankFR774584.1). Nevertheless, the ML tree showed that the 16S rDNA sequence of the Z2-4 isolate was closely related to *Bacillus* sp. (Figure 3). This result led to the assignation of this identity to isolate Z2-4 (Table 6). Finally, the strain Z2-11 was the only one classified within the Phylum Deinococcus-Thermus and associated with the species *Deinococcus sahariens*, with an identity percentage of 99.04%.

A phylogenetic analysis of the identified microorganisms was performed to support the identity obtained from Blast. Figure 3 presents the resulting phylogenetic tree obtained by the maximum likelihood method in the MEGA program. *Sulfolobus acidocaldarius* ATCC 33909 and *Zymomonas mobilis* ATCC 16S rDNA sequences were retrieved from GenBank and included in the phylogenetic analysis. The sequence of *S. acidocaldarius* was used as outgroup.

## 4. Discussion

### 4.1. Physicochemical Characterization of Thermal Water

The hot spring of Santa María Amajac is part of the Neovolcanic Axis, that crosses Mexico in a west–northwest direction [46]. Due to its mineral properties, which are considered medicinal, the thermal water from this site is used as a therapeutic agent in balneotherapy [47].

It is common in the literature to consider pH, temperature, and total dissolved solids to characterize mineral-medicinal waters. With the pH values measured in situ, it can be considered as a neutral spring (6 ≤ pH < 7.5) [48]. Nevertheless, Camargo et al. [49] conducted a study in the same area; they also carried out a physicochemical characterization of the water from Santa María Amajac and they reported a pH of 6.84, which drove them to classify the spring as slightly acidic. On the other hand, the temperature measured in situ allowed us to define the spring as containing hyperthermal waters, since it exceeds 38 °C [47], but according to the Mexican Geological Service [45], it should be classified as a hypothermal deposit. Camargo et al. [49] reported a temperature of 55 °C, which they also classified as hyperthermal. Regarding the concentration of total dissolved solids measured in situ, according to Davis [50], its waters can be classified as brackish waters (1000–10,000 ppm), and as slightly saline waters (1000–3000 ppm), according to Krieger’s classification [51]. Finally, it can be classified as mineral water (1000–3500 ppm), according to Pazdro and Kozerski [52] in Porowski [53].

In various studies with thermal waters, pH and temperature values similar to those obtained in the present study have been reported, although total dissolved solids are not always included. Rupasinghe et al. [54] studied six sources of thermal waters in Sri Lanka; they reported pH values between 6.25 and 8.35, thus classified as acidic and alkaline, and a temperature range of 39–59 °C. Chan et al. [55] analyzed various sources of thermal water in Malaysia: Semenyih, with a temperature range of 40–50 °C and a pH of 6.9; Ayer Hangast, with temperatures between 40 and 50 °C and a neutral pH of 7.1; and Dusun Tua, with temperatures of 55–75 °C and a pH of 7. Lee et al. [56] studied five thermal water sites in the Republic of Korea, two of which resemble the present study: Baegam, with a temperature of 45.9 °C and a pH of 8.93, and Dongnae, with a temperature of 59.4 °C and a pH of 7.6. On the other hand, Prieto-Barajas et al. [57] conducted a study in each season of the year in two thermal springs in Michoacán, Mexico (Tina and Bonita); it should be noted that these areas are also located in the Transversal Volcanic Axis. As for the spring sampling, the same season in which samples were taken in this study, a temperature of 66 °C and a pH of 7.64 were reported in Tina, while a temperature of 45 °C and a pH of 7.03 were reported in Bonita. Finally, Nabi Najar et al. [58] analyzed a thermal water source located in India, in the Borong area, for which they reported a temperature of 52.3 °C, a pH of 5.32, and a concentration of total dissolved solids of 1330 mg/L. It is worth mentioning that the thermal water sites mentioned above have similarities with the temperature and pH parameters found in the present study; in the case of the work of Nabi Najar et al. [58], the total dissolved solids also resemble those reported in Santa María Amajac.

### 4.2. Hydrogeochemical Analysis

In this study, the physicochemical analysis showed that Z2 thermal water can be classified as sulfate-calcic water, as described above. However, Camargo et al. [49] classified the water from Santa María Amajac as calcium-sodium-sulfate-chloride, with values of 0.09 mg/L for carbonates, 250.60 mg/L for bicarbonates, 236.00 mg/L for chlorides, 486.40 mg/L for sulfates, 48.20 mg/L for magnesium, 8.10 mg/L for potassium, and 128 mg/L for sodium. The thermal waters from the present study were reported with lower values, as shown in Table 2, with only the magnesium value being similar to that reported by Camargo et al. [49]. Despite both of these thermal water sources being in the same area, there are differences in their values and classification.

The dissolution capacity of minerals from Figure 2 in decreasing order is portlandite > halite > thenardite > brucite > mirabilite > epsomite > anhydrite > gypsum. According to Bratcher et al. [59], this could be attributed to the ionic strength of the water. With this association, it is possible that calcium mainly originates from portlandite > anhydrite, while sulfate is likely to arise from thenardite > mirabilite > epsomite > gysum, which can be attributed to the water pH and temperature in the study area. The same authors mention that carbonate minerals usually react geochemically (through dissolution) due to ionic strength, rather than due to the acidity of the water (pH). On the other hand, they point out that minerals of volcanic origin usually correlate their geochemistry with pH (the lower the pH, the greater the dissolution).

Camargo et al. [49] pointed out that, in the study site, thermal water emanating in limestones and travertine deposits over fracture surfaces and dissolution caverns, favorable conduits for hot water circulation toward the surface.

Although the mineralogy of the study area still needs to be confirmed, it is anticipated that the predominant rock groups (calcareous, clastic, and volcanic [60]), are associated with the predominant water chemical ions (SO_4_^2−^ + Ca^2+^).

### 4.3. Characterization of the Isolated Strains

Isolated strains were morphologically characterized by cell shape recognition, Gram staining, and endospore production. Z1 and Z2 yielded 94% of isolates corresponding to rod shape, with the remaining 6%, strain Z2-11, exhibiting a round or coccus shape. In both sites, 100% of the strains tested positive for Gram staining (Figure A1, Figure A2, Figure A3, Figure A4, Figure A5, Figure A6, Figure A7, Figure A8, Figure A9, Figure A10, Figure A11, Figure A12, Figure A13, Figure A14, Figure A15, Figure A16, Figure A17 and Figure A18, center). Lastly, it was observed that most of the strains produced endospores (Figure A1, Figure A2, Figure A3, Figure A4, Figure A5, Figure A6, Figure A7, Figure A8, Figure A9, Figure A10, Figure A11, Figure A12, Figure A13, Figure A14, Figure A15, Figure A16 and Figure A17, right), except for strain Z2-11 (Figure A18, right). Bacterial morphology is genetically determined; however, the interaction between cells and the environment can affect morphogenesis, thus contributing to prokaryotic versatility [61]. Different bacterial shapes aid cells in adapting to extreme conditions, and they may even modify their morphology to suit the environment [62].

In a study conducted by Mohammad et al. [9], isolating 10 bacterial strains from 5 thermal water sources in Jordan, it was reported that most strains were Gram-positive with the ability to produce endospores, which are similar to those isolated in this study. Kumar and Sharma [63] sampled three hot springs in the Himalayas and isolated different strains, which they characterized as bacilli with wavy margins, semi-raised elevation, and white color. These strains were also Gram-positive and spore-forming. Nabi Najar et al. [58] reported the presence of Gram-positive thermophilic bacteria with bacillus shape and spore-forming capability. Abdollahi et al. [13] isolated and characterized bacteria of various colors, mostly spore-forming and Gram-positive. These previously described results are similar to the bacteria isolated in the present study, leading to morphological characteristics analogous to bacterial microorganisms found in hot springs.

Likewise, this study observed that the strains exhibited diverse morphologies, as depicted in Figure A1, Figure A2, Figure A3, Figure A4, Figure A5, Figure A6, Figure A7, Figure A8, Figure A9, Figure A10, Figure A11, Figure A12, Figure A13, Figure A14, Figure A15, Figure A16, Figure A17 and Figure A18 (left). Additionally, Table 3 describes the diversity regarding morphological characteristics both on agar plates, slant and vertical agar tubes, and liquid media. Colonies on solid media can be defined as a visible mass formed from a mother cell; hence, characteristics such as shape, texture, elevation, pigmentation, and changes in growth due to nutrient media are important for identification [64].

Mohammad et al. [9] also conducted a morphological characterization of the isolated strains in their work and observed differences among them in terms of color, margin, shape, and texture; they described strains with gray, cream, and white colors, opaque or translucent, with a wrinkled appearance adhering to the agar surface, although no physiological characterization of the isolates was performed.

On the other hand, the physiological characterization of strains in this study at different temperature and pH ranges, as well as at different NaCl concentrations (Table 4), demonstrated colony growth, whether exhibiting higher or lower biomass or being inhibited. The production of metabolites and the optimal growth of microorganisms are related to the specificity of temperature, pH, and salinity. Unsuitable parameters can affect metabolite production and similarly inhibit growth [65]. In the work of Mohammad et al. [9], based on the water temperatures of thermal springs (39.9–60 °C), they classified strains as moderate thermophiles and thermophiles; likewise, with the pH of the sites (7.03–8.6), strains were classified as neutrophils and alkaliphiles.

Table 5 displays the results of biochemical tests for each of the isolated strains. Most of the strains fermented glucose, and only three strains fermented three sugars, albeit without gas production, and only one of these produces H_2_S. A proportion of 94% of the strains tested positive for catalase. As for hydrolysis tests, 94% of strains could hydrolyze casein, 44% starch, 83% gelatin, 77% cellulose, and only 27% of strains hydrolyzed pectin and xylan. It is worth noting that the tests performed on the isolates in this study were qualitative, so enzymatic characterization was not the focus. The importance of thermo-enzymes lies in their thermostability above 55 °C, the temperature at which the strains in this study were isolated, indicating their suitability for specific industrial processes [15].

The results of hydrolysis by the isolates are of biotechnological importance due to their applications in pharmaceutical, detergent, food and animal feed, textile, paper, leather tanning, and biorefinery industries, as the main enzymes from thermophiles include cellulases, amylases, xylanases, pectinases, proteases, esterases, phytases, and lipases, primarily due to their industrial application [66]. Enzymes are considered an eco-friendly alternative to reducing chemical catalysts, which can help to reduce the generation of toxic compounds and the use of solvents, benefiting the environment, while potentially reducing costs [67].

### 4.4. Molecular Identification of the Isolated Strains

Table 6 displays the results of molecular identification. Predominant species related to the isolates included *A*. *gonensis*, *Bacillus* sp., *B*. *licheniformis*, *B*. *tequilensis*, *B*. *subtilis*, *P*. *dendritiformis*, and *D*. *sahariens*. One strain was associated with the taxon *Aneurinibacillus* sp., while another was linked to a putative taxon. 

In the hot springs sampled by Kumar and Sharma [63], each with distinct temperatures and pH levels, in Surya Kund (87.8 °C and pH 8.5), they reported *B*. *subtilis*, *B*. *licheniformis*, *B. mycoides*, *P*. *dendritiformis*, *Paenibacillus ehimensis*, *B. simplex*, *B. thermoamylovorans*, *Geobacillus stearothermophilus*, *B. megaterium*, *Actinobacillus hominis*, and *B*. *tequilensis*. In Draupadi Kund (67 °C and pH 9.4), they isolated *B*. *subtilis*, *B. borstelensis*, *B*. *licheniformis*, *B*. *thermoamylovorans*, *G*. *stearothermophilus*, *B*. *megaterium*, *A*. *hominis*, *B*. *tequilensis*, *Brevibacillus thermoruber*, *Brevibacillus choshinensis*, and *Streptococcus pyogenes*. The last sampled site was Yamunotri Tapt Kund (46.8 °C and pH 7.5), where they isolated *B*. *subtilis*, *B*. *licheniformis*, *Streptococcus thermophilus*, *Actinobacillus seminis*, *B*. *mycoides*, *A*. *hominis*, *Brevibacillus choshinensis*, *S*. *pyogenes*, *Thiobacillus denitrificans*, *Paenibacillus thiominolyticus*, and *Lysinibacillus sphaericus*. Variation in diversity was observed across the three sites, likely influenced by temperature as a key environmental factor affecting microbial diversity.

Lee et al. [56], in South Korea, isolated 29 strains from five hot spring sources associated with the taxa *Geobacillus* sp., *Caldibacillus* sp., *Aeribacillus* sp., as well as the species *B*. *licheniformis*, and *Thermoactinomyces vulgaris*. Of these, 75.8% exhibited amylase, lipase, and protease activity.

Patel and Dudhagara [68] isolated *B*. *tequilensis* from hot springs in Maharashtra, India, with an incubation temperature of 45 °C, aiming to characterize the enzyme xylanase and to test its potential for use in rice pulp pre-bleaching. Strains Z2-2 and Z2-3 from this study were associated with *B*. *tequilensis* and showed positive xylanase activity.

As shown in Figure 3, most of the strains were classified within the Phylum Firmicutes, except for strain Z2-11, classified as Deinococcus–Thermus. The characteristics described below are derived from descriptions of various bacterial species within the Phylum Firmicutes in the Bergey’s Manual of Systematic Bacteriology [31], henceforth refer to this source.

The Phylum Firmicutes consists of 26 families and 233 genera; strains identified in the phylogenetic tree belong to the class “Bacilli”, encompassing genera such as *Bacillus*, *Anoxybacillus*, *Paenibacillus*, and *Aneurinibacillus* [31]. General features of the Phylum Firmicutes include predominance of Gram-positive species, although rare Gram-negative examples have been reported. They exhibit phenotypic diversity, and while most grow at neutral pH, some can be isolated from acidic or alkaline environments. They typically produce catalase and spores resistant to freezing and heat [31].

Figure 3 classifies strains Z1-2, Z2-2, Z2-3, Z2-4, Z2-5, Z2-6, Z2-9, and Z2-10 as different *Bacillus* species, aligning with the aforementioned general characteristics. Colonial morphology on agar plates and tubes varies among species, contrary to endospore production.

Strain Z1-1 was classified as *Anoxybacillus*, described as a Gram-positive rod with growth in a temperature range of 30–60 °C, pH 8–10, and NaCl concentrations up to 5% but not at 10%, consistent with the Table 4 results. It is also reported to exhibit no growth at 25 °C, neither at pH 5, 6, 8, and 9. Catalase production is generally observed but may vary among species. Strain Z2-1 was classified as *Aneurinibacillus*, typically identified as Gram-positive with ellipsoidal endospores. Strains Z2-7 and Z2-8 were classified under the genus *Paenibacillus*, known for being Gram-positive. Some species can be strictly aerobic, microaerophilic, facultatively anaerobic, or obligately anaerobic, mesophilic or thermophilic; they are also classified as neutrophilic and alkaliphilic and may exhibit variable or negative Gram staining, and most test positive for catalase. Spores produced by these species are typically oval. Optimal growth has been reported between 28 °C and 40 °C, with a near-neutral pH optimum, although some species have been reported as alkaliphiles and show inhibited growth at 10% NaCl concentrations. The characteristics described align with the results of this study, corroborated by Figure A14 and Figure A15, and Table 3 and Table 4.

Only the strain Z2-11 was classified as part of the genus *Deinococcus*, with the characteristics of a Gram-positive bacterium with an orange color, consistent with Figure A18. *Deinococcus sahariens* is a strictly aerobic, non-spore-forming cocci isolated from hot springs in Tunisia (Sahara). Additionally, it is known for its resistance to ultraviolet radiation. Several species in this genus share this characteristic, leading to its recognition as a radioresistant genus, exemplified by *Deinococcus radiodurans*. Optimal growth conditions have been reported in temperature ranges of 30 to 65 °C, with an optimum of 50 °C, pH range of 6–10, with an optimum of 7.5, and salt tolerance of 0–4% [69]. There are also coincidences with some of the characteristics presented by Z2-11 in Table 4. 

The strain Z2-11 is phylogenetically closer to *D. sahariens*. Both strains and the strains of *D. geothermalis* were phylogenetically closer to the archaea outgroup *S. acidocaldarius* than any other taxon included of bacteria, as expected. This could be due to some amount of horizontal genetic exchange among these taxa in their evolutionary history, since *S. acidocaldarius* thrives in similar environments.

Hot springs worldwide are of interest due to their microbial diversity. It has been demonstrated that microbial communities can be influenced by the physicochemical properties of hot springs, with temperature being a key factor affecting microbial diversity [57].

## 5. Conclusions

Mexico possesses thermal water sources within the Trans-Mexican Volcanic Axis from which thermophilic microorganisms can be isolated. The physicochemical characterization of the hot springs in this region—from which microorganisms were isolated—was made possible by this research work in the State of Hidalgo.

Morphological characterization, culturing in agar plates and tubes, as well as molecular taxonomic identification, primarily placed them within the Phylum Firmicutes, including species such as *Anoxybacillus gonensis*, *Bacillus licheniformis*, *Bacillus tequilensis*, *Bacillus subtilis*, and *Paenibacillus dendritiformis*, along with the genus *Aneurinibacillus*. Furthermore, a close taxon to *Deinococcus sahariens*, a bacterium belonging to the Phylum Deinococcus–Thermus, was isolated. Because of positive activity of enzymes like amylase, cellulase, pectinase, and xylanase, as well as positive results from qualitative testing, these isolates demonstrate potential application in biotechnological and industrial processes. Moreover, they adapt to severe environments, such as high temperatures, acidity, or alkalinity, among others. Thus, bacteria isolated from thermal waters remain a source of extremophilic microorganisms with potential industrial-scale applications.

## Figures and Tables

**Table 1:** Physicochemical parameters measured in situ.

Parameters	Units	Z1	Z2
Dissolved oxygen	mg/L	4.00	2.45
pH	pH	6.76	6.73
Temperature	°C	44.74	53.77
Electrical conductivity	μS/cm	3170	2778
Total dissolved solids	mg/L	1585	1389
Salinity	PSU	1.60	1.37
Redox	mV	226.8	248.8

**Table 2:** Physicochemical characterization of thermal water from Z2 site.

Parameters	Concentration (mg/L)
Ca^2+^	234.12
Mg^2+^	41.67
Na^+^	114.25
K^+^	11.72
PO_4_^3−^	1.87
NO_₃_^−^	0
CO_3_^2−^	0
HCO_3_^−^	203.79
Cl^−^	13.37
SO_4_^2−^	895.95
Hardness	729.23
Electrical conductivity	2651 *
Effective salinity	8.698

The hardness is the result of the sum of CaCO_3_ + MgCO_3_; * µS/cm.

**Table 3:** Morphology characterization in solid and liquid media of the isolated strains.

Morphology		Z1							Z2										
		1	2	3	4	5	6	7	1	2	3	4	5	6	7	8	9	10	11
Agar plate																			
Shape	Round	+				+			+						+		+		+
	Rhizoid		+	+	+											+			
	Amoeboid						+	+		+	+	+	+	+				+	
Margin	Undulate	+				+			+			+		+	+				
	Lobate						+	+		+	+		+			+		+	
	Entire																+		+
	Serrate		+	+	+														
Elevation	Flat	+			+	+			+	+	+	+		+	+	+	+		+
	Rough		+	+									+					+	
	Papillate						+	+											
Texture	Butyrous	+			+	+			+	+		+		+	+	+	+		+
	Membranous		+	+			+				+		+					+	
	Viscid							+											
Color	Beige	+	+			+	+	+	+					+	+		+		
	White			+	+					+	+		+			+		+	
	Yellow											+							
	Orange																		+
Agar deep tubes																			
Morphology	Papillate	+	+	+			+	+	+	+	+	+	+	+	+			+	+
	Beaded				+														
	Filiform					+										+	+		
Agar slant tubes																			
Morphology	Echinulate	+	+							+	+	+		+			+		
	Rhizoid			+			+	+					+					+	
	Filiform				+										+	+			+
	Effuse					+													
	Beaded								+										
Liquid media																			
Superficial growth	Membranous	+	+	+		+	+		+	+		+	+	+			+	+	
	Flocculent				+			+								+			
	Ring										+								
	No growth														+				+
Turbid	Light		+				+	+	+		+		+			+			+
	Medium														+				
	Null																+		
	Transient	+		+	+	+				+		+		+				+	
Sediment	Viscid	+	+			+		+		+	+	+	+		+	+		+	
	Lumpy			+			+												
	Granular				+				+					+			+		+
Quantity of sediment	Abundant	+								+					+			+	
	Scanty		+	+	+	+	+	+	+		+	+	+	+		+	+		+

+: Growth characteristic.

**Table 4:** Temperature and pH ranges for growth and tolerance tests of the isolated strains.

Tolerance Test		Z1							Z2										
		1	2	3	4	5	6	7	1	2	3	4	5	6	7	8	9	10	11
T	25 °C	--	*	*	*	*	*	*	*	*	*	*	*	*	*	*	*	*	*
	37 °C	*	*	+	+	*	+	+	+	+	+	*	+	+	*	+	+	+	*
	50 °C	*	+	+	+	*	+	+	*	+	+	*	+	*	*	*	*	*	*
	60 °C	*	+	+	+	*	+	+	*	+	+	*	+	+	*	*	--	*	-
pH	5	--	--	--	--	°	°	--	°	°	°	°	°	°	°	°	°	--	*
	6	--	--	--	°	°	°	--	*	°	*	*	+	*	°	*	+	+	*
	7	*	+	+	+	*	+	*	°	*	+	*	*	*	*	°	°	°	*
	8	--	*	*	*	°	*	°	°	°	°	*	*	°	°	°	°	°	°
	9	--	+	+	+	*	+	+	+	*	+	*	+	+	+	+	°	*	°
NaCl	2%	*	+	+	+	*	*	+	+	+	+	*	+	+	*	*	°	+	-
	5%	*	+	+	+	--	+	+	+	+	+	°	°	+	°	*	+	+	*
	7%	°	+	°	+	°	+	+	°	°	°	°	°	°	°	°	°	°	--

T (temperature); no growth (--); inhibited growth (-); scanty biomass (°); medium biomass (*); abundant biomass (+).

**Table 5:** Biochemical tests of the isolated strains.

Biochemical Test		Z1							Z2											Control	
		1	2	3	4	5	6	7	1	2	3	4	5	6	7	8	9	10	11	C-	C+
F	Glucose	-	-	+	+	-	+	+	+	+	+	-	+	+	+	+	+	+	-	+	+
	Lactose	-	-	+	-	-	-	-	-	-	-	-	+	-	+	-	-	-	-	+	+
	Sucrose	-	-	+	-	-	-	-	-	-	-	-	+	.	+	-	-	-	-	+	+
P	H_2_S	-	-	-	-	-	-	-	-	-	-	-	-	-	+	-	-	-	-	-	-
	Gas	-	-	-	-	-	-	-	-	-	-	-	-	-	-	-	-	-	-	+	-
A	Indole	-	-	-	-	-	-	-	-	-	-	-	-	-	-	-	-	-	-	+	-
	Catalase	-	+	+	+	+	+	+	+	+	+	+	+	+	+	+	+	+	+	-	+
H	Casein	+	+	+	+	+	+	+	+	-	+	+	+	+	+	+	+	+	+	-	+
	Starch	-	+	-	-	-	+	+	+	+	-	-	+	+	-	-	-	+	-	-	+
	Gelatin	-	+	+	+	+	+	+	+	+	+	-	+	+	+	+	+	+	-	-	+
	Cellulose	-	+	+	+	-	+	+	+	+	+	-	+	+	+	+	+	+	-	-	-
	Pectin	-	-	+	+	-	+	+	-	-	-	-	+	-	-	-	-	-	-	-	-
	Xylan	-	-	-	-	-	-	-	+	+	+	-	-	+	-	-	-	+	-	-	-

F (Sugar fermentation); P (Production); A (Activity); H (Hydrolysis); *Escherichia coli* (C-); *Bacillus subtilis* (C+), - (Negative result); + (Positive result).

**Table 6:** Taxonomic characterization of the isolated strains.

Strain	Bp	Identity	Gen Bank Accession Number	Per Ident	Query Cover	E Value
Z1-1	1428	*Anoxybacillus gonensis* strain G2, complete genome	PP579944	99.86%	99%	0.0
Z1-2	1397	*Bacillus licheniformis* strain Ba2 16S rRNA	PP579945	100%	100%	0.0
Z1-3	1400	*Bacillus licheniformis* strain ZBT15 16S rRNA	PP579946	97.34%	99%	0.0
Z1-4	1396	*Bacillus licheniformis* strain ASMK2 16S rRNA	PP579947	97.86%	100%	0.0
Z1-5	1418	*Bacillus licheniformis* strain ASMK2 16S rRNA	PP579948	97.46%	99%	0.0
Z1-6	1422	*Bacillus licheniformis* strain RFNB2 16S rRNA	PP579949	97.40%	100%	0.0
Z1-7	1398	*Bacillus licheniformis* strain TB212 16S rRNA	PP579950	100%	100%	0.0
Z2-1	1383	*Aneurinibacillus* sp. Bac270 16S rRNA	PP579951	99.78%	100%	0.0
Z2-2	1403	*Bacillus tequilensis* strain SQA-76 16S rRNA	PP579952	100%	100%	0.0
Z2-3	1400	*Bacillus tequilensis* strain KHJP1 16S rRNA	PP579953	97.43%	100%	0.0
Z2-4	1384	*Bacillus* sp. L-bf-R2A-30.3 16S rRNA	PP579954	99.78%	99%	0.0
Z2-5	1416	*Bacillus licheniformis* strain Ba2 16S rRNA	PP579955	99.22%	100%	0.0
Z2-6	1396	*Bacillus subtilis* strain ZG13-41 16S rRNA	PP579956	100%	100%	0.0
Z2-7	1399	*Paenibacillus dendritiformis* strain ClaCZ2 16S 16S rRNA	PP579957	100%	100%	0.0
Z2-8	1422	*Paenibacillus dendritiformis* strain ClaCZ203 16S 16S rRNA	PP579958	99.44%	100%	0.0
Z2-9	1419	*Bacillus licheniformis* strain KaK2A 16S rRNA	PP579959	99.37%	100%	0.0
Z2-10	1403	*Bacillus subtilis* strain 3667 16S rRNA	PP579960	99.93%	100%	0.0
Z2-11	1350	*Deinococcus sahariens* strain HAN23 16S 16S rRNA	PP579961	99.04%	100%	0.0

## Data Availability

The raw data supporting the conclusions of this article will be made available by the authors on request.
